# Predictors of mothers’ postpartum body dissatisfaction based on demographic and fertility factors

**DOI:** 10.1186/s12884-020-03501-x

**Published:** 2021-01-05

**Authors:** Mehrri Tavakoli, Seyedeh Batool Hasanpoor-Azghady, Leila Amiri Farahani

**Affiliations:** Department of Midwifery and Reproductive, Nursing Care Research Center (NCRC), School of Nursing and Midwifery, Iran University of Medical Sciences, Rashid Yasemi st., Valiasr St, Tehran, 1996713883 Iran

**Keywords:** Body dissatisfaction, Postpartum, Body image, Demographic factors, Fertility factors

## Abstract

**Background:**

There are fundamental and rapid changes in body shape during pregnancy, some of which persist for an extended time after delivery and may cause dissatisfaction with body shape. Therefore, we conducted this study to determine predictors of body dissatisfaction at six months postpartum based on demographic and fertility factors.

**Methods:**

This cross-sectional study was conducted on 300 women who referred to seven health centers affiliated with Iran University of Medical Sciences, Tehran, Iran. The sampling was multistage and we collected data from a demographic and fertility questionnaire and Cooper’s Body Shape Questionnaire (BSQ-34). The independent t-test, Mann-Whitney U test, chi-square test, one-way ANOVA, Kruskal-Wallis, Pearson correlation coefficient, and multiple linear regression were used for data analysis. The level of significance was set at *P* < 0.05.

**Results:**

The mean age of participating women was 29.77 (standard deviation: 5.9) years. Body dissatisfaction had a statistically significant association with variables such as body mass index (BMI) at six months postpartum, gestational age, the receipt of information about body shape, spouse’s views on the shape of a woman’s body, and mode of delivery. These variables predicted 34% of body dissatisfaction based on multiple linear regression.

**Conclusion:**

Postpartum body dissatisfaction is related to a several variables. Paying attention to these variables will help to plan and improve postpartum counseling and educational programs.

## Background

In today’s society, there is tremendous emphasis on physical attractiveness and fitness. Social pressures on women to have a slim body and meet the standards of beauty have increased women’s dissatisfaction with their physical appearance [1]. During pregnancy, there are fundamental and rapid changes in a woman’s body shape and weight. Some changes begin far before a woman is aware of her pregnancy [2]. Society expects women to control their weight after child birth, which is stressful [3]. Longitudinal studies that examine body image during pregnancy show that although pregnant women may have a period of relative satisfaction about their body image during mid- to late pregnancy, worrying about it may peak after delivery [3–5]. Overall, all prospective studies that have investigated the effect of body image on postpartum depression found a positive association between body dissatisfaction and postpartum depression at different time periods, up to 12 months after delivery [1, 6]. In pregnancy, women tolerate body changes (e.g., large abdomen, swollen feet, weight gain) because they perceive the positive function of these changes [3]; however, body dissatisfaction is reported by many women during the postpartum period [7].

The results of a systematic review showed that women who had better body image were more likely to breastfeed [8] and that women who breastfeed have a stronger relationship with their infants [9]. On the other hand, women who were more concerned about their bodies were less likely to breastfeed or stopped breastfeeding earlier [8]. Some women stopped breastfeeding because of their disgust with breast shape after giving birth [10]. Body dissatisfaction has also been reported to reduce sexual satisfaction [11]. Swedish women often found it difficult to adapt to physical changes and expressed discontent with the looseness of their genitals and the size of their breasts [12]. The reactions to such changes depend on a variety of factors, including cultural and family attitudes, social factors such as visual media, and social normative pressures, standards, and definitions of social beauty [13].

Among the body image factors reported during the postpartum period, younger women were reported to have more body satisfaction than older women because they had lower weight and fewer children, and paid more attention to themselves [14]. Body image satisfaction was also associated with college education, employment, regular exercise, receipt of postpartum information, childcare assistance, the presence of a positive attitude about oneself, and the husband’s positive attitude towards his wife [14].

The results of studies showed different findings on women’s attitudes towards their postpartum body image. In one study, mothers from Minnesota (USA) reported that body dissatisfaction escalated from one to nine months after childbirth [15]. Erbil et al. [14] also reported that 42.3% of Turkish women had a negative attitude about their body size. However, 57% of African-American women had a positive attitude about their body size after childbirth and 79.1% said that their spouses also had a positive attitude towards their body size [4].

Although there are physiological changes that occur during pregnancy and the postpartum period that put women under sociocultural pressures [3, 4], only a few studies have used a qualitative approach to assess postpartum body dissatisfaction [1]. To date, no studies have assessed postpartum body dissatisfaction among Iranian women; therefore, we conducted this study to determine predictors of body dissatisfaction based on demographic and fertility factors at six months postpartum in Iranian women.

## Methods

This cross-sectional study enrolled 300 women, six months after childbirth, from seven health centers affiliated with Iran University of Medical Sciences. The university covers seven districts in Tehran (the capital of Iran). The sampling was multi-stage. First, a health center was randomly selected from each district and we determined the number of eligible individuals as a quota from this center. Next, continuous sampling was conducted at each center between May 2018 and January 2019. The inclusion criteria for study participation consisted of the ability to read and write, lack of stressful events in the previous six months, no use of drugs or any medications related to mental disorders, lack of chronic systemic disease in the mother, lack of high-risk pregnancy (except for premature labor), lack of abnormality in the child, no specific postpartum diet, and no postpartum abdominal cosmetic surgery.

We used G * power software to determine the required sample size. Sample size at 95% confidence level with test power of 0.9, effect size medium level based on Cohen’s classification and considering that the number of predictor variables was 19 of variables, was calculated to be 272 people, which in this study we considered 300 people.

Data collection tools included a demographic and fertility questionnaire and the Persian version of the 34-item Body Shape Questionnaire (BSQ-34). The BSQ-34 was developed by Cooper et al. in 1987. The BSQ-34 measures concerns about body shape and has been used to assess body dissatisfaction [16]. Each item is measured with a six-point Likert scale, as follows: 1 (never), 2 (rarely), 3 (sometimes), 4 (often), 5 (very often), and 6 (always). The score of the items ranges from 34 to 204. A higher score indicates greater dissatisfaction with body shape [16].

In the Rosen et al. study [17], the reliability of Cooper’s body shape questionnaire was reported as 0.88 using the test-retest method. According to Evans and Dolan [18], the internal consistency of the questionnaire was confirmed by a Cronbach’s alpha value of 0.97%. In Iran, Sadeghi et al. reported a Cronbach’s alpha value of 0.95% and test-retest of 0.82 [19].

We initiated sampling at the health centers after receipt of study approval by the Ethics Committee of Iran University of Medical Sciences (code: IR.IUMS.REC 1395. 9,311,373,008). The participants provided written informed consent for study participation. Next, participants completed the self-administered questionnaires. Data were analyzed using the independent t-test, Mann-Whitney U test, one-way ANOVA, Kruskal-Wallis, Pearson correlation, and multiple linear regression using SPSS version 21. The multiple linear regression model was used to determine the simultaneous effect of the variables, each of which was related to the dependent variable using the Enter method. Before implementation of multivariate analysis, we checked the regression assumptions, such as normality of the residuals, homoscedasticity, multicollinearity, and independence of the residuals. The significance level for all tests was *p* < 0.05.

## Results

According to the results, participants had a mean [±SD] age of 29.77 [±5.9] years. Mean [±SD] weight before pregnancy was 62.35 [±10.81] kg and six months postpartum was 66.95 [±11.54] kg. Also, participants had a mean [±SD] BMI of 23.91 [±4.1] before pregnancy and 25.69 [±4.52] six months postpartum. Most subjects had a college education (49.7%) and a relatively favorable economic status (62.7%). A total of 62% of participants were primipara. Table 1 provides additional demographic information and fertility characteristics of the participants.

**Table 1:** Demographic and fertility characteristics in women six months postpartum (*n* = 300)

Characteristics	N (%)
**Woman’s education**	
< High school	55 (18.3)
High school	96 (32)
Academic	149 (49.7)
**Spousal’s education**	
< High school	58 (19.3)
High school	109 (36.3)
Academic	133 (44.3)
**Birthplace**	
Urban	222 (74)
Rural	78 (26)
**Woman’s occupation**	
Housewife	231 (77)
Employed	69 (23)
**Spousal’s occupation**	
Employee	105 (35)
Free	148 (49.3)
Worker	38 (12.7)
Unemployed	9 (3)
**Economic status**	
Favorable	96 (32)
Relatively favorable	188 (62.7)
Undesirable	16 (5.3)
**Infertility history**	
Yes	20 (6.7)
No	280 (93.3)
**Abortion history**	
Yes	54 (18)
No	246 (82)
**Mode of delivery**	
^a^NVD	139 (46.3)
^b^C/S	161 (53.7)
**Parity**	
Primipara	186 (62)
Multipara	114 (38)
**Pregnancy**	
Wanted	281 (93.7)
Unwanted	19 (6.3)
**Wanted and unwanted baby’s gender**	
Wanted	225 (75)
Unwanted	75 (25)
**Gestational age**	
Term	281 (93.7)
Preterm	19 (6.3)
**Breastfeeding period**	
< 6 Months	35 (11.7)
6 Months	265 (88.3)
**Nutrition status of the infant**	
Breast-feeding	213 (71)
Bottle-feeding	36 (12)
Both	51 (17)
**Receiving information about the body shape**	
yes	94 (31.3)
No	206 (68.7)
**Spouse’s views on the shape of a woman’s body**	
Very satisfied	97 (32.3)
Somewhat satisfied	138 (46)
Indifferent	37 (12.3)
Somewhat dissatisfied	21 (7)
Very dissatisfied	7 (2.3)

^a^Normal Vaginal Delivery

^b^Caesarean section

Pearson correlation coefficient showed that body dissatisfaction had a directly significant relationship with BMI at six months postpartum (*p* < 0.001; r = 0.45). But there was no relationship between body dissatisfaction with women’s age (*P* = 0.80; r = − 0.14). Tables 2 and 3 show the relationship between other demographic and fertility variables with body dissatisfaction score**.**

**Table 2:** The relationship between demographic characteristics and body dissatisfaction score in women six months postpartum (*n* = 300)

Characteristics	Mean (SD)	***P***-value
**Woman’s education**		
< High school	74.29 (32.43)	0.832^*^
High school	73.48 (33.62)	
Academic	76.07 (34.01)	
**Spousal’s education**		
< High school	76.91 (32.94)	0.587^*^
High school	72.28 (29.99)	
Academic	76.2 (36.48)	
**Birthplace**		
Urban	77.70 (33.89)	0.281^**^
Rural	66.99 (31.30)	
**Woman’s occupation**		
Housewife	73.41 (34.90)	0.273^**^
Employed	79.96 (31.23)	
**Spousal’s occupation**		
Employee	79.03 (37.42)	0.389^***^
Free	74.16 (31.75)	
worker	65.47 (25.83)	
Unemployed	79.11 (38.59)	
**Economic status**		
Favorable	73.80 (32.59)	0.372^***^
Relatively favorable	76.69 (34.60)	
Undesirable	63.31 (25.23)	

^*^ One way ANOVA ^**^Independent t-test ^***^Kruskal–Wallis

**Table 3:** The relationship between fertility characteristics and body dissatisfaction score in women six months postpartum (*n* = 300)

Characteristics	Mean (SD)	***P***-value
**Infertility history**		
Yes	76.50 (33.53)	0.795^*^
No	74.80 (33.57)	
**Abortion history**		
Yes	82.59 (32.36)	0.061^**^
No	73.23 (33.59)	
**Mode of delivery**		
^c^NVD	69.45 (28.28)	0.007^**^
^d^C/S	79.63 (36.89)	
**Parity**		
Primipara	74.40 (34.56)	0.731^**^
Multipara	75.75 (31.87)	
**Pregnancy**		
Wanted	74.53 (33.49)	0.158^*^
Unwanted	80.63 (33.21)	
**Wanted and unwanted baby’s gender**		
Wanted	74.16 (33.29)	0.504^**^
Unwanted	77.16 (34.21)	
**Gestational age**		
Term	73.61 (32.59)	0.029^*^
Preterm	94.21 (41.40)	
**Breastfeeding period**		
< 6 Months	80.23 (40.81)	0.401^**^
6 Months	74.21 (32.46)	
**Nutrition status of the infant**		
Breast-feeding	72.01 (30.96)	0.081^***^
Bottle-feeding	88.89 (42.96)	
Both	77.18 (34.31)	
**Receiving information about the body shape**		
yes	83.38 (36.19)	0.003^**^
No	71.05 (31.56)	
**Spouse’s views on the shape of a woman’s body**		
Very satisfied	56.34 (24.15)	< 0.001^***^
Somewhat satisfied	79.78 (30.74)	
Indifferent	86.73 (35.60)	
Somewhat dissatisfied	93.76 (35.73)	
Very dissatisfied	117.43 (53.62)	

^c^Normal Vaginal Delivery

^d^Caesarean section

*Mann-Whitney U **Independent t-test ***Kruskal–Wallis

We placed the participants in the two groups of normal BMI and high BMI. The chi-square test results showed that there was no statistically significant difference between the two groups of normal BMI and high BMI in terms of receipt of information about body shape (*p* = 0.84).

An examination of the one-to-one relationship of variables to body dissatisfaction indicated a statistically significant relationship between variables, which included BMI at six months postpartum, gestational age, receipt of information about body shape, spouse′s views on the shape of a woman′s body, and the mode of delivery. These variables were entered into the multiple linear regression model using the Enter method and they could remain within the model. According to the results, the mean score for body dissatisfaction of women at six months postpartum for each unit added to the BMI increased by 2.52 units; however, this amount decreased by 8.08 units in women with NVD compared to women who underwent cesarean section. The mean score for body dissatisfaction among subjects who gave birth to term infants decreased by 15.28 units compared to those who gave birth to preterm infants. This score increased by 8.77 units in subjects who received body shape information compared to those who did not receive any information. Furthermore, from the spouse’s view on the shape of a woman’s body, the mean score of body dissatisfaction in women whose spouses were somewhat satisfied with their body shape was 14.54 units higher than those whose spouses were very satisfied with their body shape. In women whose spouses were indifferent to their body shape, the mean score was 23.6 units higher than women whose spouses were very satisfied with their body shape. In women whose spouses were somewhat dissatisfied with their body shape, the mean body dissatisfaction score was 28.62 units higher than women whose spouses were very satisfied with their body shape, while in women whose spouses were very dissatisfied with their body shape, the mean body dissatisfaction score was 41.60 units higher than the women whose spouses were very satisfied with their body shape.

Therefore, based on the results, 34% of changes in dependent variables (body dissatisfaction at six months postpartum) were explained by independent variables that included BMI at six months postpartum, gestational age, receipt of information about body shape, spouse’s views on the shape of a woman’s body, and the mode of delivery. Among these variables, the six-month postpartum, BMI was the most effective predictor of postpartum dissatisfaction (Table 4).

**Table 4:** Results of multiple linear regression analysis to investigate the effect of demographic and fertility characteristics on physical satisfaction

Independent variable		B Coefficient	Standardized Coefficient	Statistics	P-value	95% CI (L / H)	R2
BMI six months postpartum		2.52	0.34	6.72	< 0.001	1.78/3.26	0.34
Mode of delivery	^e^C/S	Reference Category					
	^f^NVD	−8.08	−0.12	−2.49	0.013	−14.47/− 1.69	
Gestational age	Preterm	Reference Category					
	Term	−15.28	−0.11	−2.32	0.021	−28.24/− 2.32	
Receiving information about the body shape	No	Reference Category					
	Yes	8.77	0.12	2.52	0.012	1.93/15.61	
Spouse’s views on the shape of a woman’s body	Very satisfied	Reference Category					
	Somewhat satisfied	14.54	.21	3.79	< 0.001	6.99/22.09	
	Indifferent	23.6	0.23	4.31	< 0.001	12.83/34.37	
	Somewhat dissatisfied	28.62	0.21	4.21	< 0.001	15.24/41.99	
	Very dissatisfied	41.60	0.18	3.75	< 0.001	19.78/63.42	

^e^Cesarean Section

^f^Normal Vaginal Delivery

## Discussion

The present study aimed to determine the predictors of body dissatisfaction at six months postpartum based on demographic and fertility characteristics. The results showed that body dissatisfaction had a significant relationship with variables such as BMI at six months postpartum, gestational age, receipt of information about body shape, spouse’s views on the shape of a woman’s body, and mode of delivery. Among these variables, the six-month postpartum BMI was the most effective predictor of postpartum body dissatisfaction. The higher the BMI, the greater the dissatisfaction. In this respect, the results of a study by Gjerdingen et al. indicated that an elevated BMI was the most important factor associated with body dissatisfaction [15]. Evidence suggests that increased attention to appearance, especially postpartum weight and obesity, results in increased body dissatisfaction among women [20]. The target weight of some women after childbirth is even lower than the pre-pregnancy weight, which results in increased body dissatisfaction [21]. Our study subjects weighed a mean of 4.600 kg more than their pre-pregnancy weights in the six months following delivery. Participants in the study by Gjerdingen et al. weighed 2.5 kg more in the nine months following delivery than before pregnancy [15]. Since the timing of these two studies is different, it is not possible to compare postpartum weight retention; however, in a study conducted by Rallis et al., it has been shown that dissatisfaction with body shape was most prevalent in the six months following delivery. In the study mentioned above, the mean BMI six months after delivery was 24.90; in the current study, it was 25.69, which could be attributed to the large sample size of the current study [5]. The results of another study showed that most participants described body changes during pregnancy with one objective and meaning. In their opinion, the unique event of becoming a mother enabled women to deal positively with changes in their bodies during pregnancy. Society also has a positive interpretation of pregnancy changes, but dissatisfaction and striving to reclaim the postpartum body dominated [22].

In the present study, there was no relationship between women’s age, the woman and her husband’s level of education, the woman and her husband’s job, and economic status with body dissatisfaction. Although body dissatisfaction in women whose husbands were workers was less than women whose husbands were employees, this difference was not statistically significant. In this respect, Gjerdingen et al. [15] reported that woman’s age, education, income, health insurance, and their employment status were not related to body shape dissatisfaction. It has also been noted that the relative importance of social factors regarding postpartum body dissatisfaction is uncertain and not well-studied. In another study, the results showed no relationship between age, job, and education with body shape dissatisfaction [23].

The results reported by Walker et al. on the relationship between dissatisfaction with body shape and race showed that black women were more satisfied with their body shape than white women [24]. In the current study, all subjects were of the same race; therefore, we compared body shape dissatisfaction between women in urban versus rural areas. In terms of socio-economic factors, just as blacks in most parts of the world are at a lower socioeconomic level than whites, village residents have a lower socioeconomic status than urban residents. Our findings showed that body dissatisfaction among women born in a village was lower than among those born in a city. This was not a statistically significant difference, as rural-born women made up a quarter of the subjects in our study. However, another explanation for decreased body dissatisfaction among rural women might be that they are less exposed to receipt of information, social pressures, and media publicity.

The results of the present study show that body dissatisfaction is related to the views of the spouse on the shape of the woman’s body. Women whose spouses were very satisfied with their body shape expressed higher body satisfaction. In this regard, the results of another study showed that a negative assessment by the spouse was strongly related to body dissatisfaction and, after the weight variable, the history of negative encounters in the family was of tremendous importance in terms of dissatisfaction with the shape of the body [25]. In the postpartum period, spouses play a major role in supporting women by providing positive feedback on the physical changes of the woman’s body [21]. Mickelson et al. concluded that maternal satisfaction with body shape after childbirth was accepted only with the consent of the spouse and it was indirectly related to the satisfaction of their private affairs. In other words, mothers understand that their spouses do not accept them in sexual relations, making them less satisfied with their private lives. Researchers in this study reported a clear link between body shape dissatisfaction and intimate relationships. Postpartum intimacy for a woman might be related to her husband’s satisfaction with the shape of her body [26].

Our study found that women with less information about body shape were more satisfied with their body shape. Information coupled with cultural and social pressures may make women feel unattractive, dissatisfied with their body, anxious, depressed, and stressed [4]. Most of the women’s self-consciousness with their bodies is rooted in the image of a beautiful body created by society. This self-consciousness and monitoring of the body as an item for assessment by others leads to increased body dissatisfaction [27]. By promoting a thin body as a beauty criterion, the media also causes women to experience unrealistic postpartum pressure on their body shape, and makes them want to quickly lose the weight they gained during pregnancy [21].

The present research showed a significant association between body dissatisfaction and the mode of delivery (NVD, cesarean section). In a qualitative study, Berry indicated that in women during five weeks after cesarean section, factors such as wound repair, therapeutic interventions such as injections and insertion of intravenous lines, digestive and nutritional problems, movements needed to breastfeed the baby, and reduced body function caused them to feel dissatisfied with their body image [28]. In our study, there was no association between body dissatisfaction with parity (multipara and primipara), abortion, infertility history, wanted and unwanted pregnancy, and baby’s gender. Gjerdingen et al. also reported that variables such as the baby’s gender were not related to body shape dissatisfaction after delivery [15]. In line with our study, there was no significant difference in body image between multiparous and primiparous women in other studies conducted in Iran [29]. The findings of the study by Rahmanian et al. also showed that there was no association between wanted and unwanted pregnancy and dissatisfaction with body shape; however, parity was related to body dissatisfaction. Because multiparous women have experienced pregnancy and are aware of changes in body shape during this period, they know which physical changes are reversible and which are permanent; therefore, multiparous women are more dissatisfied with their body shape than primiparous women who see pregnancy and its changes as a motherhood experience [23].

In our study, there was a statistically significant difference in the mean score of body dissatisfaction (*p* = 0.009), which depended on gestational age. Mothers who gave birth to preterm infants were more dissatisfied with their body shape. The researcher did not find a comparative study in this regard. In the current study, we observed no relationship between body satisfaction of mothers with different feeding conditions for infants (breast milk, bottle-feeding, both) and the duration of breastfeeding, which contradicted the results from other studies. The results of these studies have indicated that dissatisfaction with body shape affects breastfeeding self-efficacy, reduces the tendency to breast-feed, and causes women to stop breastfeeding earlier [8, 10, 20].

There were a number Strengths and limitations to this study. One of the strengths of this study is that we considered the variable of the spouse’s views on the shape of a woman’s body. According to Duncombe et al., women who had a positive attitude towards their bodies after childbirth often stated that their spouses also had a positive attitude about their body size [4]. Another strength is that in the present study, we examined the association of body dissatisfaction with receiving information about body shape. The results showed participants who received information were more dissatisfied about their body shape. We also examined the role of BMI as an intervening variable in this regard. T-test showed that there was no statistically significant difference in mean BMI in terms of receiving information about body shape.

The study had several limitations. Because the data were based on self-reported answers by the subjects, the response to some items might have been influenced by cultural factors and society values. A major difficulty we encountered in data analysis was how to score the BSQ-34. There was no difference between worry about body shape and its absence. Cooper et al. did not determine the cut-off point for the questionnaire. Although we observed no relationship between participants’ education and body shape dissatisfaction, education might have influenced the relationship between other variables and body shape dissatisfaction. Approximately 50% of the participants in our study had a college education; therefore, the generalization of the results of the present study might be less for studies where participants have lower educational levels. Finally, the assessment of body dissatisfaction was limited to just six months postpartum.

## Conclusion

The results showed a relationship between body dissatisfaction and several variables. The BMI at six months postpartum had the greatest effect on body dissatisfaction. Paying attention to weight and the other variables can help clinicians prepare and develop counseling or education programs for women to gain proper weight during pregnancy and have proper diet and postpartum activities.

## Data Availability

The datasets used and analyzed during the current study are available from the corresponding author on reasonable request.

## Abbreviations

BSQ: Body Shape Questionnaire
BMI: Body Mass Index
NVD: Normal Vaginal Delivery
IV line: Intravenous Line
